# Gender differentials in readiness and use of mHealth services in a rural area of Bangladesh

**DOI:** 10.1186/s12913-017-2523-6

**Published:** 2017-08-18

**Authors:** Fatema Khatun, Anita E. Heywood, Syed Manzoor Ahmed Hanifi, M. Shafiqur Rahman, Pradeep K. Ray, Siaw-Teng Liaw, Abbas Bhuiya

**Affiliations:** 1 Universal Health Coverage, Health Systems and Population Studies Division, International Centre for Diarrhoeal Disease Research, Bangladesh (icddr,b), 68 Shaheed Tajuddin Ahmed Sarani, Mohakhali, Dhaka, 1212 Bangladesh; 2 School of Public Health and Community Medicine, UNSW Australia, Sydney, NSW 2052 Australia; 3 Institute of Statistical Research and Training, University of Dhaka, Dhaka, 1000 Bangladesh; 4 General Practice Unit, South West Sydney Local Health District and Ingham Institute of Applied Medical Research, 1 Campbell Street, Liverpool, NSW 2170 Australia; 5 Partners in Population and Development (PPD), Sher-E-Bangla Nagar, Agargaon, Dhaka, 1207 Bangladesh

## Abstract

**Background:**

Traditional gender roles result in women lagging behind men in the use of modern technologies, especially in developing countries. Although there is rapid uptake of mobile phone use in Bangladesh, investigation of gender differences in the ownership, access and use of mobile phones in general and mHealth in particular has been limited. This paper presents gender differentials in the ownership of mobile phones and knowledge of available mHealth services in a rural area of Bangladesh.

**Methods:**

We interviewed 4915 randomly selected respondents aged 18 years and above. Associations between gender and knowledge of available mHealth services, use of existing mHealth services and intentions to use mHealth services in the future were examined by multivariate logistic regression analysis, controlling for the effect of categorised covariates.

**Results:**

Of the 4915 respondents to the survey, 61.8% of men (1213/1964) and 34.4% of women (1015/2951) owned a mobile phone. For men, mobile phone ownership was highest among those aged 18–29 years (*n* = 663, 76.3%), and for women among those aged 30–39 years (*n* = 825, 44.7%). A higher proportion of men owned phones compared to women, irrespective of socioeconomic status (SES) as indicated by asset index (*p* < 0.001). Although mobile phone ownership on average was lower among women, they were more likely to share their mobile phone with their family members (19.7%) compared to men (11.6%, *p* < 0.001). Greater number of men were more likely to be aware of the use of mobile phones for healthcare compared to women (38.5% vs 26.5%, *p* < 0.001). Knowledge about available mHealth services was lower among women than men; however, intention to use mHealth services in the future was high for both genders, irrespective of age, education and socioeconomic status.

**Conclusions:**

Compared to men, women are less likely to own a mobile phone and less aware of available mHealth services, despite high intention to use mHealth among both genders. To optimise the use of mHealth services and to achieve equity of use, uptake strategies should target women, with a focus on the poorer and less educated groups.

**Electronic supplementary material:**

The online version of this article (doi:10.1186/s12913-017-2523-6) contains supplementary material, which is available to authorized users.

## Background

Traditional gender roles result in women lagging behind men in the utilisation of healthcare services and this is particularly evident in developing countries [1, 2]. To achieve equitable health service use, female participation is crucial to align with Goal 5 of the United Nations’ (UN) Sustainable Development Goals (SDG) 2030: “Achieve gender equality and empower all women and girls”. The concept of mobile phone use in healthcare (mHealth) was established to assist underserved people accessing health services irrespective of time, place and person [3]. Evidence of the importance of mHealth in healthcare and public health is growing with promising results in mHealth projects globally [4–17]. The majority of mHealth projects in developing countries have targeted women and children to improve maternal, newborn and child health (MNCH), to increase immunisation coverage and to control infectious diseases. These projects contribute to the UN Millennium Development Goals 4, 5 and 6, which now will be continued with Sustainable Development Goal 3: “Ensure healthy lives and promote well-being for all at all ages” [4, 10, 14, 15, 17–24]. Utilisation of any healthcare service is associated with its physical accessibility [25, 26]; in the case of mHealth services, access to mobile phones and phone capabilities are essential. However, a study conducted in countries in Asia and Africa found women were disadvantaged in terms of access to mobile phones, use of mHealth services and mobile internet use, with an overall higher technology illiteracy barrier than men [27].

The ‘Striving and surviving: exploring the lives of women at the base of pyramid’ report by the GSMA mWomen program in 2012 found that the gender differentials in mobile phone ownership is profound in the lowest income group [27]. Due to social, economic and cultural gaps, it is now evident that worldwide compared to men, women are 21% less likely to own a mobile phone [28]. In Kenya, men were predominant in the mobile phones ownership (1.7 times) and SMS use (1.6 times) compared to women [29]. In the majority of families in developing countries, husbands are the main users and owners of mobile phones. According to the GSMA mWomen report, the majority (72%) of married women stated that their husbands would not allow them to own a mobile phone; of the married women who owned phones, 82% of them believed that it makes their husbands more suspicious. Overall, the husband is most likely to get the first mobile phone in a family of lower socioeconomic status [27]. It is therefore important to investigate the role of the husband or the head of the household in facilitating or restricting the access of women to mobile phones. A recent study in Bangladesh analysed mobile phone ownership in households from 2009 to 2011 in a rural area and showed that overall ownership is increasing over time and the equity gap is starting to dissipate [30]. However, in households in the lowest socioeconomic strata, women with primary education or jobs without formal education are least likely to own a mobile phone [30].

The importance of women being involved in decision making for healthcare-seeking has been studied previously [31, 32]. Maternal health services are underutilised in disadvantaged populations where these services are needed the most. Available healthcare services are underutilised due to economic, educational and gender power relations [33]. Ahmed et al. (2010) investigated the relationships between women’s socioeconomic status, educational qualification and empowerment status and the utilisation of maternal health services. They found that women with higher empowerment scores are more likely to use modern contraception, antenatal care, and have a skilled attendee during childbirth [33]. Lower infant and child mortality, better child nutrition, lower fertility rates and protection against abuse were observed among women of higher socioeconomic status [34–37]. It is therefore important to empower women for decision making about healthcare and encourage them to seek and utilise available services.

In recent years, coverage and reach of mobile phones have created huge potentials for reducing maternal health disparity in terms of cost and inadequate health infrastructure [27]. However, disparities in access to mobile technologies exist not only due to gender, but also due to education level, socioeconomic status and geographic location in developing countries [38, 39]. Like other developing countries, in Bangladesh uptake of mobile phones is increasing sharply and more than 20 mHealth services are available around the country [40]. It is important to explore the uptake of mHealth services among women by age group, education level and socioeconomic status. A systematic analysis of the gender gap is needed to understand community readiness for mHealth. This study aimed to examine gender differences in awareness of mobile phone use for healthcare services and knowledge of available mHealth services. Irrespective of the status of mobile ownership, the study also aimed to explore gender differences in their future intention to use mHealth. The importance and implications of the results may guide mHealth program planners and policy makers in developing mHealth policies and implementation that lead to equitable ownership and use of mHealth, in particular, strategies to enable access to mobile technology by the world’s most vulnerable women.

## Methods

### Study site, sample size and data collection

This study was conducted in a rural sub-district of Bangladesh named Chakaria situated in the south-eastern part of Bangladesh. Since 1999, International Centre for Diarrhoeal Disease Research, Bangladesh (icddr,b) has been maintaining a Health and Demographic Surveillance System (HDSS) to capture demographic events [41]. This analysis used data from a large survey conducted in the Chakaria HDSS between November 2012 and April 2013 [42]. Chakaria HDSS covers 20,124 households which is habituated by 62,458 adult households’ members. Among them, 5152 study participants (2188 adult males and 2964 adult females) were randomly chosen for this survey [43].

Sampling frames were stratified by age groups (18–29 years, 30–39 years, 40–49 years, and 50+ years) and gender (male, female). We used 50% value for sample size calculation separately for age groups and gender to estimate the proportion of respondents with knowledge of available mHealth services, using tele-consultation services and attitude towards use of mHealth in future with 95% confidence. For each age group, sample size was estimated by using the fraction (400/population of the age group with lowest population size) multiplied by the population in the age group. This formula estimated 400 sample in each age group and provided self-weighted sample size. Sample size were 766, 570, 400 and 452 for the 18–29 years, 30–39 years, 40–49 years and 50+ years age groups respectively for male. For females sample size were 1222, 785, 400 and 557 for the 18–29 years, 30–39 years, 40–49 years, and 50+ years age groups respectively. From the HDSS database, two separate files were created, one for male and another for female. From each file, four sub-files were created based on the age groups and then respondents were randomly chosen from respective age groups.

Data were collected by female interviewers through household visit. A pretested questionnaire developed in the Bangla language used for data collection (an English translation is available in the appendix: Additional file 1). Data collection process was monitored by two field supervisors. For quality assurance, field manager revisited the 5% of the household to cross check the collected information.

### Description of the mHealth services in the Chakaria HDSS service area

In Bangladesh, Grameenphone limited launched first telemedicine services in the country named Healthline 789 which covers 10 million Grameenphone subscribers. Healthline 789 is a business model for-profit telehealth program, costs USD 0.38/call for 5 min. Healthcare services provided upon dialing HealthLine 789 include access to medical information and consulting with doctors. In addition, emergency services, SMS based laboratory reports, and ambulance services also offered. A group of registered healthcare professionals are available for 24 h in a day and 7 days in a week at the physical front office (physician’s interface) [44, 45].

In 2009, Ministry of Health and Family Welfare launched mHealth services to strengthen all districts and sub-districts hospitals by providing consultation services through mobile phones. One Medical Officer is available for medical and emergency consultation services. This service is free of cost and easy for a patient to take health services from anywhere and anytime [45].

### Ethical approval

The study was approved by the Ethical Review Committee (ERC) of icddr,b and the Human Research Ethics Committee (HREC) of the University of New South Wales (UNSW), Australia. Informed written consent or thumb impression (for illiterate participants) was obtained from each of the study participants as per the standard methods of obtaining consent in Bangladesh. Confidentiality and anonymity of the data were ensured and participants' data were de-identified prior to analysis.

### Statistical analysis

The survey included questions on the sociodemographic characteristics of the respondents such as age, sex, educational qualification of the respondents and socioeconomic status of the household; technological capabilities such as receiving and sending SMS, and mobile internet capabilities; awareness of mobile phone use for healthcare; knowledge of existing mHealth services; and intentions of mHealth use. Awareness of mobile phone use for healthcare was determined based on the following inquiry: “respondent knows that mobile phone can be used for healthcare (yes/no)”. Knowledge of the largest commercial telemedicine service, named ‘HealthLine 789’, was determined by asking if the respondent knew about calling a special number for healthcare (yes/no). If the answer was yes, then the respondent was asked about the number to call. Similarly, the respondent was asked about t mHealth services provided by the Ministry of Health and Family Welfare at the Upazila Health Complex (UHC) (yes/no) and intention to use mHealth services in future (yes/no).

We used principal component factor analysis (PCA) to estimate the asset score (proxy of socioeconomic status) of the study participants by using a wealth score based on respondents household assets [45]. Ownership of following 14 assets (cupboards/almirah, table/chairs, van/rickshaw, beds, radio, television, bicycle, motorcycle, refrigerator, sofa, electric fan, sewing machine, mobile/land phone and availability of electricity at home) at household level were used to calculate asset index. Weighted scores were then divided into 5 quintiles, lowest quintile represents poorest households and highest quintile representing the wealthiest households. Bangladesh Demographic and Health Survey and other studies used this validated tool as a proxy of socioeconomic status of a household [46].

The objective of this study was to examine the association between gender and mobile phone ownership and mobile phone capabilities (SMS use and mobile internet use), awareness of mobile phone use for healthcare, knowledge of existing mHealth services and intention to use mHealth services in the future. Since some universal confounding factors such as age, education and socioeconomic status are present in the data, which may modify the relationship between gender and the outcome variables (Table 1), a separate analysis for each level of confounder was conducted to eliminate any effect on the gender and response relationship. In each case, the percentage of positive outcomes and odds ratio (OR) with 95% CI and associated *p*-values from univariate analysis, are reported. Further, multivariable logistic regression analyses were conducted that included gender and all confounders as covariates. The average predicted probability of positive outcome was then plotted separately for males and females for each level of the included variables to assess the net effect of gender after adjusting for the effect of each confounder. For this analysis, four multivariable logistic regression models were employed with the dependent and independent variables listed in Table 1. We used statistical software STATA version 10.0 for this analysis.

**Table 1:** Dependent and independent variables used in multivariable logistic regression models

	Model 1	Model 2	Model 3	Model 4
Dependent Variables	Awareness about use of mobile phone for healthcare (Yes = 1, No = 0)	Knowledge of ‘HealthLine 789’ (Yes = 1, No = 0)	Knowledge of government mHealth services at Upazila Health Complex (Yes = 1, No = 0)	Intention to use mHealth services in future (Yes = 1, No = 0)
Independent variables	Gender	Gender	Gender	Gender
	Age	Age	Age	Age
	Education	Education	Education	Education
	Socioeconomic status	Socioeconomic status	Socioeconomic status	Socioeconomic status

## Results

A total of 4915 respondents (1964 males, 2951 females) were interviewed through household visits. During day time males were absent, thus our interviewers revisited household up to three times to maximize the response rate. Overall response rate was 95% (4915/5152), 89.8% males and 99.6% female were interviewed.

### Mobile phones ownership and technological capabilities of the respondents

Overall, 2228 (45%) respondents owned a mobile phone. However, ownership was significantly higher among males (61.8%) than females (34.4%; *p* < 0.001). Mobile phone ownership was higher among males aged 18–29 years (76.3%) and ownership among males decreased for age groups 40 years and above. Among females, ownership was higher among the 30–39 year age group (44.7%) and ownership decreased to 17.3% in the 50 years and above age group. Mobile phone ownership was higher among males compared to females in low income households (31.2% vs 13.3%; *p* < 0.001) and also in the higher income households (83.2% vs 61.3%; *p* < 0.001) (Table 2). Forty-four per cent of males with no education had a mobile phone compared to 23.4% of women with no education (*p* < 0.001). The gender gap between mobile phone ownership decreased when education level was higher; for example, women with an education level of 11 or more years had a similar rate of ownership to men (91.1% vs 88.6%; *p* = 0.063) (Table 2).

**Table 2:** Ownership of mobile phones among males compared to females, obtained from bivariate analysis in a household survey in rural Bangladesh (*N* = 4909)

Variables	Gender	Number of respondents (N)	% of respondents with mobile phone	*p*-value
Age (years)				
18–29	Male	633	76.3	*p* < 0.001
	Female	1155	35.5	
30–39	Male	518	74.1	*p* < 0.001
	Female	825	44.7	
40–49	Male	380	54.7	*p* < 0.001
	Female	414	33.6	
50+	Male	430	31.8	*p* < 0.001
	Female	554	17.3	
Education (years of schooling)				
None	Male	883	44.4	*p* < 0.001
	Female	1452	23.4	
1–5 years	Male	658	71.3	*p* < 0.001
	Female	804	36.2	
6–10 years	Male	332	82.2	*p* < 0.001
	Female	636	52.4	
11 + years	Male	88	88.6	*p* = 0.638
	Female	56	91.1	
Socioeconomic status (asset index)				
Poorest	Male	330	31.2	*p* < 0.001
	Female	675	13.3	
2nd	Male	384	54.4	*p* < 0.001
	Female	588	23.9	
3rd	Male	434	64.5	*p* < 0.001
	Female	566	35.7	
4th	Male	409	69.4	*p* < 0.001
	Female	542	42.2	
Richest	Male	404	83.2	*p* < 0.001
	Female	577	61.3	

Among the mobile phones owners (*n* = 2228), 7.3% of men and only 1.4% of women had access to and used the internet capabilities on their phones. Voice message use was around 25% in both groups (Table 3). A higher proportion of females shared their mobile phone with their family members compared to their male counterparts (19.7% vs 11.6%; *p* < 0.001) (Table 3).

**Table 3:** Technological capabilities of mobile phone owners by gender in a household survey in rural Bangladesh (*N* = 2228)

Mobile phone owner	Share mobile phoneN (%)	Voice messages useN (%)	Internet use N (%)
Female (*N* = 1015)	200 (19.7)*	257 (25.3)	14 (1.4)
Male (*N* = 1213)	141 (11.6)	314 (25.9)	89 (7.3)*
Total (*N* = 2228)	341 (15.3)	571 (25.6)	103 (4.6)

*p < 0.001

### Awareness of mobile phone use in healthcare

**Fig. 1:**
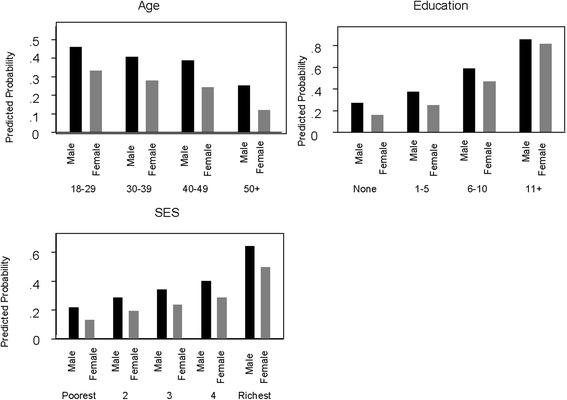
Predicted probability of awareness of mHealth services by gender, adjusted for age, education and SES

Awareness about use of mobile phones for healthcare was higher among males than their female counterparts (38.5% vs 26.5%) in general (Additional file 2: Appendix Table S1). When the relationship was adjusted for age, education and socioeconomic status, males were more aware compared to females (OR 1.74; 95% CI 1.55–1.98; *p* < 0.001). Males were more aware than females of the same age, education level and SES except for respondents who had an education level greater than 11 years. When the effect of gender was adjusted for age, the likelihood of awareness of males was 1.4 times higher in the 18–29 years age group, 1.8 times higher in the 30–39 years age group, 2.6 times higher in the 40–49 years age group and 3.2 times higher in the 50+ years age group, compared to their female counterparts. Similarly, uneducated males were 2.2 times more aware of the use of mobile phones for healthcare than uneducated females (Additional file 2: Appendix Table S1). The relationship between male gender and awareness of the use of mobile phones in healthcare was statistically significant in most of the categories for education level except for an education level of 11 years and above. Similar findings were observed when the association between gender and awareness was adjusted by SES. Males from any SES category were more aware of the use of mobile phones in healthcare than females in the same category. The association between gender and awareness was adjusted for all other covariates in a multivariable model showed that male who had education of 6 years and higher, and came from higher SES were more likely aware about use of mHealth services (Table 4). When the association between gender and awareness was adjusted for all other covariates in a multivariable model, the predicted probability of having awareness about use of mHealth services was higher among males than females (Fig. 1). For example, the predicted probability for males from lower income households was 0.22 while it was only 0.13 for females of the same SES. Similarly, males from a higher income household have a higher average predicted probability than females from the same SES (Fig. 1).

**Table 4:** Awareness of mHealth services among males compared to females, adjusted for age, education and SES in a household survey in rural Bangladesh, (*N* = 4909)

Variables	Adjusted OR (95% CI)	*p*-value
Gender		
Male	1.9 (1.6–2.1)	<0.001
Age (years)		
18–29	1.0	
30–39	0.9 (0.8–1.1)	0.415
40–49	0.9 (0.7–1.1)	0.176
50+	0.4 (0.3–0.5)	<0.001
Education (years of schooling)		
None	1.0	
1–5 years	1.2 (1.0–1.5)	0.010
6–10 years	2.4 (2.0–2.8)	<0.001
11+ years	7.4 (4.6–12.0)	<0.001
Socioeconomic status (asset index)		
Poorest	1.0	
2nd	1.4 (1.1–1.7)	0.006
3rd	1.7 (1.4–2.1)	<0.001
4th	2.0 (1.6–2.6)	<0.001
Richest	4.2 (3.3–5.3)	<0.001

### Knowledge of existing mHealth services

**Fig. 2:**
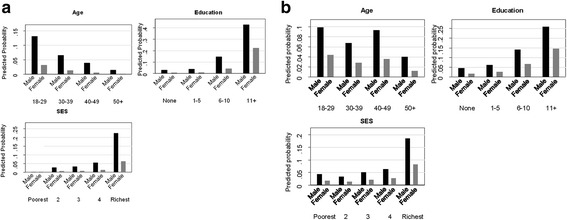
**a** Predicted probability of having knowledge about calling for commercial mHealth services – HealthLine 789 by gender, adjusted for age, education and socioeconomic status**.** **b** Predicted probability of having knowledge about calling for Government mHealth services at Upazila Health Complex by gender, adjusted for age, education and socioeconomic status

The results for knowledge of available mHealth services shows that only 7.0% of males and 1.7% of females were aware of the commercial mHealth service called HealthLine 789; and 7.6% of males and 3.1% of females were aware of government mHealth services. Although a very low percentage of the respondents knew of existing mHealth services, the likelihood of knowing about the services was 4 times higher in the case of HealthLine 789 and 2.5 times higher for government mHealth services among males compared to females. When the association between gender and responses was adjusted for other sociodemographic characteristics, males were more likely to be aware of the HealthLine 789 and the government mHealth services (Additional file 2: Appendix Table S2). Similar results were observed when the association between gender and knowledge was adjusted for all other covariates (for HealthLine789: OR 5.9, 95% CI 4.1–8.9, *p* < 0.001; for government services: OR 2.6, 95% CI 2.0–3.4, *p* < 0.001) (Table 5). The corresponding average predicted probability was higher for males than females from the same for age, SES,and education background (Fig. 2a and b).

**Table 5:** Knowledge about calling available mHealth services HealthLine 789 and government mHealth services (UHC), adjusted for age, education and socioeconomic status in a household survey in rural Bangladesh

	HealthLine 789		Government mHealth services at UHC	
Variables	aOR (95% CI)	*p*-value	aOR (95% CI)	*p*-value
Gender				
Male	5.9 (4.1–8.5)	<0.001	2.6 (2.0–3.4)	<0.001
Age (years)				
18–29	1.0		1.0	
30–39	0.5 (0.3–0.7)	<0.001	0.7 (0.5–1.0)	0.087
40–49	0.2 (0.1–0.4)	<0.001	1.1 (0.8–1.6)	0.629
50+	0.1 (0.0–0.1)	<0.001	0.4 (0.2–0.6)	<0.001
Education (years of schooling)				
None	1.0		1.0	
1–5 years	0.7 (0.4–1.1)	0.118	1.1 (0.7–1.6)	0.605
6–10 years	1.9 (1.2–3.1)	0.005	2.1 (1.4–3.1)	<0.001
11+ years	7.9 (4.5–13.9)	<0.001	3.0 (1.8–5.2)	<0.001
Socioeconomic status (asset index)				
Poorest	1.0		1.0	
2nd	13.1 (1.7–100.1)	0.013	0.7 (0.4–1.3)	0.275
3rd	13.8 (1.8–104.3)	0.011	1.0 (0.6–1.8)	0.890
4th	19.8 (2.7–147.5)	0.004	1.2 (0.7–2.0)	0.458
Richest	73.1 (10.0–534.6)	<0.001	3.3 (2.0–5.3)	<0.001

### Intention to use mHealth services

**Fig. 3:**
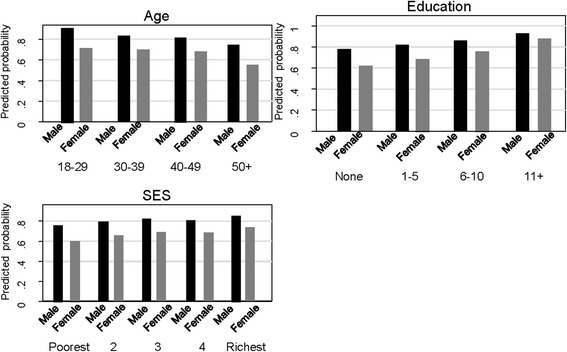
Predicted probability of intention to use mHealth in the future by gender, adjusted for age, education and socioeconomic status

In total, 81.4% of males and 67.5% of females showed their interest to use mHealth services in near future. Males were twice as likely to intend to use mHealth services in the future than females (*p* < 0.001). These findings were similar irrespective of age, education or socioeconomic status, except for women with 11 years or more of education (Additional file 2: Appendix Table S3). The association between gender and intention to use mHealth in the future was adjusted for all other covariates in a multivariable model and showed that males who had an education of 6 years or more, and with highest SES were more likely have intentions to use mHealth services in the future (Table 6). The predictive probability of using mHealth in the future was high (more than 0.50) for males and females. However, probability was higher in males in each sub-group of sociodemographic characteristics (Fig. 3).

**Table 6:** Intention to use mHealth in the future, adjusted for age, education and socioeconomic status in a household survey in rural Bangladesh

Variables	Adjusted OR (95% CI)	*p*-value
Gender		
Male	2.1 (1.8–2.4)	<0.001
Age (years)		
18–29	1.0	
30–39	1.0 (0.8–1.2)	0.862
40–49	1.0 (0.8–1.2)	0.918
50+	0.6 (0.5–0.7)	<0.001
Education (years of schooling)		
None	1.0	
1–5 years	1.1 (1.0–1.3)	0.108
6–10 years	1.5 (1.2–1.8)	<0.001
11+ years	3.0 (1.7–5.5)	<0.001
Socioeconomic status (asset index)		
Poorest	1.0	
2nd	1.1 (0.9–1.4)	0.193
3rd	1.3 (1.1–1.6)	0.007
4th	1.3 (1.0–1.5)	0.025
Richest	1.5 (1.2–1.8)	0.001

## Discussion

This study identified significant gender-based differentials in mobile phone ownership, knowledge of available mHealth services and relative contributors to the digital divide. The study findings highlight three key issues: 1) ownership of mobile phones is lower among women, particularly women of low socioeconomic status and low education; 2) awareness of mobile phone use in healthcare was low among women and women had lower use than men across all socioeconomic strata and education levels; and 3) the predictive probability of using mHealth in the future was higher than the current use level for both genders. Men with 5 or more years of education and those from the richest households were more likely to have higher intention to use mHealth in future compared to females with the same education and SES.

Access to mobile phones among target users is key for successful mHealth services implementation and uptake. Ownership of mobile phones is higher among males in Bangladesh and this is also observed in other developing countries [27, 29]. The gap between ownership was not found among men and women of highest education levels. Similar observations were also observed in Kenya [29]. Education empowers women and results in increased ownership of a mobile phone, as mobile phone capabilities are directly related to education. Although literature suggested that the gender gap is lower among people of higher SES [27], in this study, gender differentials existed in all socioeconomic strata. This may be due to cultural contexts where women do not enjoy freedom to enter robust communication platforms such as mobile phones and the internet because of inherent suspicion about women communicating freely. Although there is a natural trickle-down effect of technology to disadvantaged groups, to have this happen for women without any special intervention may be quite slow. If lack of money is the only barrier for women to own a mobile phone then providing a phone to women on credit or instalment could be a possibility. The other possibility is promoting the use of mobile phones through microfinance programs where there are 18.3 million members who can use the phone for economic reasons [47]. It is also important that mHealth programs focusing on maternal and child health find out the leverage mechanism to provide the target group with access to handsets and ensure their active participation. Inequalities in mobile phone access and low maternal health services utilisation were observed in Nigeria [48]. To increase access to target users, one option is to create a mechanism for men to share their phones with female family members. However, our study showed that men are less likely than women to share their mobile phones with other family members [27, 31, 49]. For the health condition where patient privacy and confidentiality is crucial sharing phone is not appropriate solution. In such case, health program should provide cheap and toll free mobile phone services for poor women. Toll free mobile phone services has shown effective utilization in MNCH care in Bangladesh [17]. To increase female involvement in the mHealth, mobile phone access is not the only barrier. There are several barriers identified such as health literacy, user friendly mHealth services. Before implementing mHealth program in developing countries, it is important to examine the level and degree of health literacy among the end users which will help to design mHealth program. Voice messaging through mobile phones are interactive, simple and the most appropriate strategy to communicate with the patient with low health literacy level. The Aponjon project of Bangladesh highlighted the importance of designing mHealth interventions that are appropriate for the end-user with low health literacy [50]. To overcome technological and literacy barrier, text messages and its content should be follow human centred-design and tailored to meet the user need [46, 51].

To utilise any healthcare services, people need to have knowledge of its availability and accessibility. Knowledge of mHealth services was low (31%) in general and gender differences existed irrespective of education and socioeconomic status. Women with high education and those of higher socioeconomic status also have low knowledge of mHealth services, which indicates that available mHealth services are not focused appropriately for women to be aware about the mHealth program. Bangladesh is pioneering girls’ education and is on track to achieve Millennium Development Goal 2 to promote gender equality and empower women [23]. There is evidence that women usually bear primary responsibility for family care and health. Although there is a lack of mobile phone ownership, low awareness of mobile phone use in healthcare and low knowledge about available mHealth services, there is a high probability of women in every socioeconomic and education group being interested in joining the mHealth program in future. Data showed that the gender gap in ownership decreases when women are educated. Thus there is a need for an inter-sectoral approach to educate women and empower them.

### Strengths and limitations

This is the first study in Bangladesh to report on gender differentials in mHealth use. It was conducted in a HDSS site located in a rural sub-district, and is thus generalisable to the other rural areas of Bangladesh. This study used two separate sampling frames, one for males and another for females, which were stratified by age group, allowing males and females to be compared separately. Large sample size and higher response rate increased the precision of the results. This study was conducted in a rural area of Bangladesh so it may not be generalisable to urban settings or countries with different gender inequalities.

## Conclusions

Compared to men, women are less likely to own a mobile phone and are less aware of available mHealth services. Both genders intend to use mHealth services in future. To optimise the use of mHealth services and to achieve equity of use, uptake strategies should target women with a focus on the poorer and less educated groups.

## Additional files

Additional file 1: Survey questionnaire. (DOC 572 kb)

Additional file 2: Appendix Table S1. describes awareness about use of mobile phone for healthcare among males compared to females adjusted for other covariates for age, education and socioeconomic status. **Appendix Table S2.** describes knowledge of existing mHealth services among males compared to females, adjusted for other covariates for age, education and SES; and **Appendix Table S3.** describes intention to use mHealth services in future among males compared to females, adjusted for other covariates for age, education and socioeconomic status (DOCX 25 kb)

## Abbreviations

HDSS: Health and Demographic Surveillance System
mHealth: Mobile health
MNCH: Maternal, Newborn and Child Health
OR: Odds Ratio
SDG: Sustainable Development Goals
UHC: Upazila Health Complex
UN: United Nations
