# Supplementary material for: Gender differentials in readiness and use of mHealth services in a rural area of Bangladesh
Source: BMC Health Serv Res. 2017 Aug 18;17:573. doi: 10.1186/s12913-017-2523-6 (PMC5563057; doi:10.1186/s12913-017-2523-6)
Supplement: Supplementary file 1 — Survey questionnaire. (DOC 572 kb) [file 12913_2017_2523_MOESM1_ESM.doc]

**Future health system with mobile phone based healthcare: an exploration of the**

**potential and challenges in Chakaria, Bangladesh**

**Health Seeking Behaviour and mobile phones uses of Villagers**

HH Number: ÑÑÑÑÑÑÑÑÑÑÑÑÑÑÑÑÑÑ Respondent’s ID: ÑÑÑÑÑÑÑÑÑÑÑÑÑÑÑÑÑÑÑÑÑÑÑÑ

| Member number | Name | mobile phones  1= yes  2= no | Sex | Age  (yrs) | Education (completed years) | Did you get sick in the past 14 days? | How many days ago did you get sick? | Are you / patients cured today? | How many days ago were you cured? | Whom did you contact to for treatment? |
| --- | --- | --- | --- | --- | --- | --- | --- | --- | --- | --- |
|  |  |  | M-1  F-2 |  |  | Yes--1 No --2  Absent-3, DK-7 | -----days | Yes---1  No ---2 | ----- days | MBBS—1 , VD(allopathic) -2, VD (homeo) -3, SACMO/MA/CHCP-4, RLG/ Herbal-5, None -6 |
|  |  |  | M-1  F-2 |  |  | Yes--1 No --2  Absent-3, DK-7 | -----days | Yes---1  No ---2 | ----- days | MBBS—1 , VD(allopathic) -2, VD (homeo) -3, SACMO/MA/CHCP-4, RLG/ Herbal-5, None -6 |
|  |  |  | M-1  F-2 |  |  | Yes--1 No --2  Absent-3, DK-7 | -----days | Yes---1  No ---2 | ----- days | MBBS—1 , VD(allopathic) -2, VD (homeo) -3, SACMO/MA/CHCP-4, RLG/ Herbal-5, None -6 |
|  |  |  | M-1  F-2 |  |  | Yes--1 No --2  Absent-3,DK-7 | -----days | Yes---1  No ---2 | ----- days | MBBS—1 , VD(allopathic) -2, VD (homeo) -3, SACMO/MA/CHCP-4, RLG/ Herbal-5, None -6 |
|  |  |  | M-1  F-2 |  |  | Yes--1 No --2  Absent-3,DK-7 | -----days | Yes---1  No ---2 | ----- days | MBBS—1 , VD(allopathic) -2, VD (homeo) -3, SACMO/MA/CHCP-4, RLG/ Herbal-5, None -6 |

1. **Healthcare through phones**

| **1.1** | Did you ever call a doctor for medical treatment by mobile phone? | Yes | 1 | 2.1 |
| --- | --- | --- | --- | --- |
|  |  | No | 2 |  |
| **1.2** | Who did you call? | Village doctor (allopathic) | 1 |  |
|  |  | Village doctor (homeopathic) | 2 |  |
|  |  | Call centre (..............) | 3 |  |
|  |  | Other telemedicine centre | 4 |  |
|  |  | Known MBBS doctor | 5 |  |
|  |  | MBBS doctor referred by others | 6 |  |
|  |  | ICDDR,B doctor | 7 |  |
|  |  | SACMO/MA/ CHCP | 8 |  |
|  |  | TRCL | 9 |  |

**2. Cost of healthcare:** (Allopathic only)

| **2.1** | What was the healthcare related cost of the above mentioned episode? | Medicine: |  | 4.6 |
| --- | --- | --- | --- | --- |
|  |  | Consultation fees: |  |  |
|  |  | Conveyance: |  |  |
|  |  | Diagnostics: |  |  |
|  |  | Hospital: |  |  |
|  |  | Phone: |  |  |
|  |  | Total cost: |  |  |
|  |  | Don’t know | 777 |  |
|  |  | N/A | 999 |  |

**3. Medicine**

| **3.1** | What types of medicines were prescribed? | Tablet |  |  |
| --- | --- | --- | --- | --- |
|  |  | Capsule |  |  |
|  |  | Syrup |  |  |
|  |  | Powder |  |  |
|  |  | Injection |  |  |
|  |  | Others--------------------------------- |  |  |
| **3.2** | The medicine prescribed for how many days? | -----------------days | |  |
| **3.3** | Did you buy all the prescribed medicines? | All | 1 |  |
|  |  | Some | 2 |  |
|  |  | None | 3 |  |
| **3.4** | Did you take all the prescribed medicines? | Yes, all | 1 |  |
|  |  | Some all and some partial | 2 |  |
|  |  | All partial | 3 |  |
|  |  | None | 4 |  |

**4. Satisfaction with healthcare consultations and uses of mobile phones**

**A) Visit:**

| **4.1** | Did you visit a doctor? | Yes | | 1 |  |
| --- | --- | --- | --- | --- | --- |
|  |  | No | | 2 |  |
| **4.2** | Did the patient go to see the doctor?? | Yes | | 1 |  |
|  |  | No | | 2 |  |
| **4.3** | In case you visited a doctor, did you find it useful? | No useful at all | | 1 | 4.5  4.5  4.6 |
|  |  | Not so useful | | 2 |  |
|  |  | Useful | | 3 |  |
|  |  | Very useful | | 4 |  |
|  |  | N/A | | 9 |  |
| **4.4** | Why did you find it useful? | The doctor can directly observe the patients/ direct treatment cures the patients faster | | 1 |  |
|  |  | Can explain the condition to the doctor | | 2 |  |
|  |  | Consultation and prescription after diagnostics | | 3 |  |
|  |  | The doctor is known and nearby | | 4 |  |
|  |  | Can receive quick treatment and medicine | | 5 |  |
|  |  | Others ------------------------------- | |  |  |
| **4.5** | Why did not you find it helpful? | It takes more time to wait in queue | | 1 |  |
|  |  | Did not cure | | 2 |  |
|  |  | Doctor could not identify the disease | | 3 |  |
|  |  | Others ------------------------------ | |  |  |
| **4.6** | In the near future if you get sick, would you visit doctors? | Yes | | 1 | 4.8  4.8 |
|  |  | No | | 2 |  |
|  |  | Don’t know | | 7 |  |
| **4.7** | If yes, to whom would you prefer? (in order of preference) | Village Doctor (allopathic)-------------1 | 1st |  |  |
|  |  | Village Doctor (homeopath)----------- 2 | 2nd |  |  |
|  |  | MBBS -------------------3 | 3rd |  |  |
|  |  | TRCL doctor -----------4 | 4th |  |  |
|  |  | SACMO/MA/ CHCP -5 |  |  |  |
|  |  | Traditional / religious healers ------------- ----6 |  |  |  |
|  |  | Others ------------------------ | |  |  |

**B. Mobile phone ownership, usage, and related expenditure:**

| **4.8** | Do you have your own mobile phone? | Yes | | | 1 | 4.25 |
| --- | --- | --- | --- | --- | --- | --- |
|  |  | No | | | 2 |  |
| **4.9** | Can you please tell me the brand name of your mobile phone |  | | | |  |
| **4.10** | How much did you pay for the handset? | **--------------------------------** taka | | | |  |
| **4.11** | How long have you been using your own mobile phone? | Years | | |  |  |
|  |  | Months | | |  |  |
| **4.12** | What type of payment system you using? | Pre-paid | | | 1 |  |
|  |  | Post paid | | | 2 |  |
| **4.13** | Where do you charge your mobile phone? | Own house | | | 1 |  |
|  |  | At others house | | | 2 |  |
|  |  | At shop | | | 3 |  |
|  |  | Others | | |  |  |
| **4.14** | To which operator are you subscribed? | Grameen | | | 1 |  |
|  |  | Banglalink | | | 2 |  |
|  |  | Citicell | | | 3 |  |
|  |  | Warid | | | 4 |  |
|  |  | Teletalk | | | 5 |  |
|  |  | Robi | | | 6 |  |
|  |  | Other operator | | |  |  |
| **4.15** | Do you use internet on your phone? | Yes | | | 1 |  |
|  |  | No | | | 2 |  |
| **4.16** | Do you share your phone with others? | Yes | | | 1 |  |
|  |  | No | | | 2 |  |
| **4.17** | Does any member of your household own a mobile phone? | Yes | | | 1 | 4.20 |
|  |  | No | | | 2 |  |
| **4.18** | How many mobile phones are owned by members of your household? | ------------ | | | |  |
| **4.19** | What is the average purchase price of the handsets owned by others in your household? | **-------------------------** taka | | | |  |
| **4.20** | Do you know you can get SMS through mobile phones? | Yes | | | 1 |  |
|  |  | No | | | 2 |  |
| **4.21** | Do you know you can send SMS through mobile phones? | Yes | | | 1 |  |
|  |  | No | | | 2 |  |
| **4.22** | Do you read SMS? | Yes | | | 1 | 4.24 |
|  |  | No | | | 2 |  |
| **4.23** | If no, Why? |  | | |  | 4.26 |
|  |  |  |  |  |  |  |
|  |  |  |  |  |  |  |
| **4.24** | How many SMS (messages) did you receive yesterday (including all)? | -------------- | | |  |  |
|  |  | Not interested | | | 66 |  |
|  |  | Don’t know | | | 77 |  |
| **4.25** | Do you read them? | Yes | | | 1 |  |
|  |  | No | | | 2 |  |
| **4.26** | Do you use / listen to the voice messages in your phone? | Yes | | | 1  2 |  |
|  |  | No | | |  |  |
| **4.27** | Number of incoming call, SMS and voice message received per week related to | Incoming call | SMS | Voice messages | |  |
|  | 1. Health care |  |  |  | |  |
|  | 1. Agriculture |  |  |  | |  |
|  | 1. Weather forecast |  |  |  | |  |
|  | 1. Others |  |  |  | |  |
|  | 1. Don’t Know | 7 | 7 | 7 | |  |

**5. Knowledge about mobile phones use in health care**

| **5.1** | Do you know that you can seek health care through mobile phones? | Yes | 1 | 6.1 |
| --- | --- | --- | --- | --- |
|  |  | No | 2 |  |
| **5.2** | Who are using mobile phones for healthcare? | Upper class | 1 |  |
|  |  | Middle class | 2 |  |
|  |  | Lower class | 3 |  |
|  |  | Educated people | 4 |  |
|  |  | To whom has connection with doctors | 5 |  |
|  |  | Busy people / people who are in job | 6 |  |
|  |  | All type of people | 7 |  |
|  |  | Others------------------------------- |  |  |
| **5.3** | How do the people use mobile phones for seeking health care? | To call MBBS doctor | 1 |  |
|  |  | To call village doctors | 2 |  |
|  |  | To call different operators health hotline | 3 |  |
|  |  | To dial 789 | 4 |  |
|  |  | To call hospitals /clinics | 5 |  |
|  |  | To get lab test report | 6 |  |
|  |  | To call TRCL | 7 |  |
|  |  | Others------------------------------ |  |  |
| **5.4** | What are the possible uses of mobile phones for health care? | For consultation | 1 |  |
|  |  | For serial number | 2 |  |
|  |  | To track doctors ( home / chamber ) | 3 |  |
|  |  | For knowing the dose and administration of medicine | 4 |  |
|  |  | Others ----------------------------- |  |  |
| **5.5** | How many people living around you use mobile phones for heath care? | % | |  |
| **5.6** | Do Village Doctors use mobile phones for healthcare as a provider? | Yes | 1 | 5.8  5.8 |
|  |  | No | 2 |  |
|  |  | Don’t know | 7 |  |
| **5.7** | Why do they (VDs) use mobile phones? | For healthcare services | 1 |  |
|  |  | For intermediate advices | 2 |  |
|  |  | For providing immediate treatment | 3 |  |
|  |  | For giving treatment at night | 4 |  |
|  |  | For taking advices from MBBS doctors | 5 |  |
|  |  | For purchasing medicine from pharmaceuticals | 6 |  |
|  |  | For referring hospitals / clinics to the patient | 7 |  |
|  |  | Others ------------------------------ |  |  |
| **5.8** | For what purpose the village doctors use his/her mobile phones? | For contact with MBBBS doctor in case of referral | 1 |  |
|  |  | For personal use | 2 |  |
|  |  | to | 3 |  |
|  |  | For treatment | 4 |  |
|  |  | To buy medicines from pharmaceuticals | 5 |  |
|  |  | Others ------------------------------ |  |  |

**6. Uses of mobile phones for health care**

**(a) Uses of mobile phones**

| **6.1** | Do you know that you can call the doctor in some specific number using a mobile phone for medical treatment if needed? | Yes | | 1 | 6.15 |
| --- | --- | --- | --- | --- | --- |
|  |  | No | | 2 |  |
| **6.2** | To which number? |  | | |  |
| **6.3** | To which operator’s (company) number? |  | | |  |
| **6.4** | Did you ever make a phone call to this number? | Yes | | 1 | 6.15 |
|  |  | No | | 2 |  |
| **6.5** | For which type of sickness did you call to this number | For side effects of medicines | | 1 |  |
|  |  | For stomach ache | | 2 |  |
|  |  | others | |  |  |
|  |  | Not applicable | | 9 |  |
| **6.6** | After calling, what advice did you get for your sickness? | To directly visit the doctors chamber | | 1 | 6.11 |
|  |  | No advices | | 2 |  |
|  |  | Others | |  |  |
|  |  | Do not know | | 7 |  |
| **6.7** | Did you take the advice? | Yes | | 1 | 6.10 |
|  |  | No | | 2 |  |
| **6.8** | Did the advice(s) help you? | Yes | | 1 |  |
|  |  | No | | 2 |  |
| **6.9** | How did it help you? |  | |  |  |
|  |  |  |  |  |  |
|  |  |  |  |  |  |
| **6.10** | Why didn’t you take the advice you received on the phone? | I was too sick to go to the doctors chamber | | 1 |  |
|  |  |  |  |  |  |
|  |  |  |  |  |  |
|  |  |  |  |  |  |
| **6.11** | In the future, will you ever seek medical advice from the number you called before? | Yes | | 1 | 6.13 |
|  |  | No | | 2 |  |
| **6.12** | Why will you ask for medical services? | The services are good | | 1 |  |
|  |  |  |  |  |  |
|  |  |  |  |  |  |
|  |  |  |  |  |  |
|  |  |  | |  |  |
| **6.13** | Why won’t you ask for medical services through your mobile phone? | The doctor does not answer, the number was busy | | 1 |  |
|  |  |  |  |  |  |
|  |  |  |  |  |  |
| **6.14** | In the past 14 days, how many times have you called to this number? | -------- | | |  |
| (L) Phone call | | | | | |
| **6.15** | In case you called a doctor over phone, did you find it useful? | No useful at all | | 1 | 6.17  6.17  6.18 |
|  |  | Not so useful | | 2 |  |
|  |  | Useful | | 3 |  |
|  |  | Very useful | | 4 |  |
|  |  | N/A | | 9 |  |
| **6.16** | Why did you find it useful? | Got to know the doctors location | | 1 |  |
|  |  | Receiving fast and immediate treatment | | 2 |  |
|  |  | No expenditure for conveyance | | 3 |  |
|  |  | Chance to receive healthcare at anytime | | 4 |  |
|  |  | Others-------------------------- | |  |  |
| **6.17** | Why didn’t you find it as useful? | Didn’t get good treatment | | 1 |  |
|  |  | Didn’t cure | | 2 |  |
|  |  | The doctor does not help, he/she refers to other doctors | | 3 |  |
|  |  | Too expensive | | 4 |  |
|  |  | Others -------------------------- | |  |  |
| **6.18** | In the future, will you ever seek medical advice through mobile phone? | Yes | | 1 | 6.21  6.22 |
|  |  | No | | 2 |  |
|  |  | Didn’t know about healthcare through mobile phones | | 7 |  |
| **6.19** | To which doctors would you call? (according to respondent’s preference ) | Village doctor allopathic------1 | 1st |  |  |
|  |  | Village doctor (homeo) - -----2 | 2nd |  |  |
|  |  | MBBS --------------------------3 | 3rd |  |  |
|  |  | TRCL doctor -------------------4 | 4th |  |  |
|  |  | SACMO/MA/CHCP---------- 5 |  |  |  |
|  |  | Traditional / religious healer -6 |  |  |  |
|  |  | Others¨ ------------------------ | |  |  |
| **6.20** | Please tell me three main reasons for calling the doctors over phone. | Expenditure is reduced | | 1 |  |
|  |  | Saves time | | 2 |  |
|  |  | Can receive quick treatment | | 3 |  |
|  |  | No cost for transport | | 4 |  |
|  |  | Doctor is known | | 5 |  |
|  |  | When disease is severe | | 6 |  |
|  |  | If there is no one at home | | 7 |  |
|  |  | Can get treatment a midnight | | 8 |  |
|  |  | Can get to know if the doctor is in his chamber | | 9 |  |
|  |  | Know the dose and administration of medicine | | 10 |  |
|  |  | Can understand the health related problems | | 11 |  |
|  |  | others ------------------------- | |  |  |
| **6.21** | Please tell me three main reasons for not calling the doctors over phone. | Mobile phone related | | |  |
|  |  | Don’t know which number to call | | 1 |  |
|  |  | Didn’t know we could get healthcare through mobile phones | | 2 |  |
|  |  | Feel shy to talk to male doctors on the phone | | 3 |  |
|  |  | There is no need to call as the doctor’s chamber is nearby | | 4 |  |
|  |  | Doctor does not receive the calls | | 5 |  |
|  |  | It is better to consult with the doctor directly | | 6 |  |
|  |  | Don’t have a mobile phone | | 7 |  |
|  |  | Others--------------------------------- | |  |  |
|  |  | **Treatment related** | | |  |
|  |  | Don’t understand the medical condition correctly | | 8 |  |
|  |  | Can’t take physical examination | | 9 |  |
|  |  | Can get the treatment, but not the medicine | | 10 |  |
|  |  | Can’t remember the name of the medicine/can’t write | | 11 |  |
|  |  | Can’t remember the dose and administration of medicines | | 12 |  |
|  |  | Told to bring the patient to the chamber | | 13 |  |
|  |  | The doctor will ignore | | 14 |  |
|  |  | Will not/does not recognize the patient | | 15 |  |
|  |  | Doctor is busy | | 16 |  |
|  |  | Others--------------------------------- | |  |  |

**(M) SMS related**

| **6.22** | Did you ever get any messages from the government regarding national immunization day or other health related event? | Yes | 1 | 6.25 6.25 6.25 |
| --- | --- | --- | --- | --- |
|  |  | No | 2 |  |
|  |  | Could not understand/read SMS | 3 |  |
|  |  | Don’t have a mobile phone | 4 |  |
| **6.23** | After getting the message did you utilize the opportunity? | Yes | 1 | 6.25 |
|  |  | No | 2 |  |
| **6.24** | If yes, what was it? |  |  |  |
|  |  |  |  |  |
|  |  |  |  |  |
|  |  |  |  |  |
|  |  |  |  |  |
| **6.25** | Do you know that you can call the Upazilla Health Complex (UHC) for health care? | Yes | 1 |  |
|  |  | No | 2 |  |
| **6.26** | Did you ever seek health care advice from UHC over phone? | Yes | 1 | 6.28  6.28 |
|  |  | No | 2 |  |
|  |  | Don’t have a mobile phone | 3 |  |
| **6.27** | What type of health care you have received? |  |  |  |
|  |  |  |  |  |
|  |  |  |  |  |
|  |  |  |  |  |
| **6.28** | Did you ever spend money for health care through mobile phone? | Yes | 1 | 7.1 |
|  |  | No | 2 |  |
| **6.29** | How much you spend for health care seeking over phone per month? | ------------------------ taka |  |  |

**7. Preference between physical visits versus telephone consultation**

| **7.1** | In the near future ,if a member of your family gets sick at midnight what will you do?(one answer applicable) | | | | |  |
| --- | --- | --- | --- | --- | --- | --- |
|  | will bring the village doctor | 1 | Age | Female | Male |  |
|  | Will send someone to get the village doctor’s advice | 2 | <17 years |  |  |  |
|  | Will take him/her to the village doctor | 3 |  |  |  |  |
|  | Will take him/her to an MBBS doctor | 4 |  |  |  |  |
|  | Will call a telemedicine center | 5 |  |  |  |  |
|  | Will take the patient to a hospital/clinic | 6 | 18+years |  |  |  |
|  | Will call the village doctor at midnight for advice | 7 |  |  |  |  |
|  | Will take the patient to a hospital if it is severe | 8 |  |  |  |  |
|  | Will not take him/her anywhere | 9 |  |  |  |  |
|  | Will take the patient in the morning | 10 |  |  |  |  |
|  | Will take the patient to the SACMO /bring him/her | 11 |  |  |  |  |

8. Attitude toward seeking healthcare through mobile phones

| **8.1** | What do you find receiving healthcare from the doctor through mobile phones? | Not useful at all | 1 | 8.3  8.3 |
| --- | --- | --- | --- | --- |
|  |  | Not so useful | 2 |  |
|  |  | Useful | 3 |  |
|  |  | Very useful | 4 |  |
| **8.2** | Why do you find it useful? | Can get quick treatment | 1 |  |
|  |  | If the patient condition is severe | 2 |  |
|  |  | Can get quick advice | 3 |  |
|  |  | No cost for transportation | 4 |  |
|  |  | Takes less time | 5 |  |
|  |  | Less expenses | 6 |  |
|  |  | Can get treatment at night | 7 |  |
|  |  | Others ------------------------- |  |  |
| **8.3** | Why didn’t you find it useful? | **Mobile phone related** | |  |
|  |  | Cannot remember everything on the phone | 1 |  |
|  |  | Cannon remember the names of the medicines | 2 |  |
|  |  | Feel uneasy talking to the doctor on the mobile phone | 3 |  |
|  |  | Don’t trust treatment through | 4 |  |
|  |  | Don’t have the doctor’s phone number | 5 |  |
|  |  | Doctor can’t explain the disease on the phone | 6 |  |
|  |  | Others------------------------------ |  |  |
|  |  | **Healthcare related** | |  |
|  |  | The disease cures faster with direct treatment | 7 |  |
|  |  | Doctors chamber is nearby | 8 |  |
|  |  | The doctor prescribes medicines without seeing the patient | 9 |  |
|  |  | The disease cures faster with direct treatment | 10 |  |
|  |  | Do not have to give the fees | 11 |  |
|  |  | Cannot do diagnostics | 12 |  |
|  |  | Others------------------------------ |  |  |
| **8.4** | If a member of your family is sick, would you get treatment through the mobile phone, or would you go to the village doctor? | Yes | 1 | 8.6  8.7 |
|  |  | No | 2 |  |
|  |  | Depends on the situation | 3 |  |
| **8.5** | Why you would like healthcare through mobile phones? | Can get quick treatment | 1 |  |
|  |  | Can get treatment at home | 2 |  |
|  |  | Less expenses | 3 |  |
|  |  | Have to return home after visiting the doctor at night | 4 |  |
|  |  | No expenses for transportation | 5 |  |
|  |  | When called, the doctor comes home to see the patient | 6 |  |
|  |  | Saves time | 7 |  |
|  |  | Don’t have to pay fees | 8 |  |
|  |  | Others------------------------------ |  |  |
| **8.6** | Why you wouldn’t like? | **Mobile phone related** | |  |
|  |  | Doctor does not/will not give attention over the phone | 1 |  |
|  |  | Cannot explain the disease over phone | 2 |  |
|  |  | Don’t have a mobile phone | 3 |  |
|  |  | Don’ trust healthcare from mobile phones | 4 |  |
|  |  | Told to bring the patient to the chamber instead of consultation through mobile phones | 5 |  |
|  |  | Don’t know /not aware about the health care through mobile phones | 77 |  |
|  |  | Others----------------------------- |  |  |
|  |  | **Treatment related** | |  |
|  |  | Can’t examine the patient | 6 |  |
|  |  | Directly contact with village doctors | 7 |  |
|  |  | The disease cures faster with direct treatment | 8 |  |
|  |  | The doctor prescribes medicines without seeing the patient | 9 |  |
|  |  | Doctor busy / | 10 |  |
|  |  | Could not remember the name of medicine | 11 |  |
|  |  | Get prescription not the medicine | 12 |  |
|  |  | Others----------------------------- |  |  |
| **8.7** | When would you call the doctor over phone? | Serious medical condition | 1 |  |
|  |  | Get sick at night | 2 |  |
|  |  | If guardian allow | 3 |  |
|  |  | N/A | 9 |  |
|  |  | Others------------------------------ |  |  |
| **8.8** | Suppose there is a health program that sends SMS or phone calls for health care, would you be interested in joining this program? | Yes | 1 | 8.11 |
|  |  | No | 2 |  |
| **8.9** | If yes, what type of services are you expecting? | Health education | 1 |  |
|  |  | Medical consultation | 2 |  |
|  |  | Diagnosis and treatment | 3 |  |
|  |  | Medication dose related | 4 |  |
|  |  | Adherence to medication | 5 |  |
|  |  | Others--------------------------- |  |  |
| **8.10** | If yes, what type of services are you expecting? | Child health | 1 |  |
|  |  | Maternal health | 2 |  |
|  |  | Immunization | 3 |  |
|  |  | Family planning | 4 |  |
|  |  | Chronic disease | 5 |  |
|  |  | Any diseases | 6 |  |
|  |  | Others-------------------------- |  |  |
| **8.11** | If q.9.8 is no, why are you not interested? | I don’t like to participate in new treatment plans | 1 |  |
|  |  | Mobile phone related | 2 |  |
|  |  | I don’t trust medical treatment through mobile phones | 3 |  |
|  |  | Diseases cure faster through direct treatment | 4 |  |
|  |  | The doctor prescribes medicines without even seeing the patient | 5 |  |
|  |  | I feel insecure talking to the doctor via mobile phones | 6 |  |
|  |  | Need permission from husband | 7 |  |
|  |  | Others------------------------- |  |  |
|  |  | N/A | 9 |  |

**9. Food security**

| **9.1** | a) Does any member of this household ever starve due to scarcity of food? | Yes | 1 | 10.1 |
| --- | --- | --- | --- | --- |
|  |  | No | 2 |  |
| **9.2** | b) How often? | Often | 1 |  |
|  |  | Sometimes | 2 |  |
|  |  | Not always | 3 |  |

**10. Socio-demographic characteristics**

| **10.1** | Ownership of assets | Yes | No | Ownership of assets | | | Yes | | No |
| --- | --- | --- | --- | --- | --- | --- | --- | --- | --- |
|  | Electricity connection | 1 | 2 | Sewing machine | | | 1 | | 2 |
|  | Cupboard | 1 | 2 | Watch/ wall clock | | | 1 | | 2 |
|  | Table | 1 | 2 | TV | | | 1 | | 2 |
|  | Chair | 1 | 2 | Cabinet | | | 1 | | 2 |
|  | 1. Bed | 1 | 2 | Showcase | | | 1 | | 2 |
|  | Radio | 1 | 2 | Fan | | | 1 | | 2 |
|  | Bicycle | 1 | 2 | VCD/CD player | | | 1 | | 2 |
|  | Motor cycle | 1 | 2 | Refrigerator | | | 1 | | 2 |
|  | Phone | 1 | 2 | Sofa set | | | 1 | | 2 |
|  |  |  |  |  |  |  |  |  |  |
| **11** | Is there a menial laborer in this household? | | Yes | | | | 1 | |  |
|  |  |  | No | | | | 2 | |  |
| **12** | Education of HH head: | | Formal (completed years) | | | |  | |  |
|  |  |  | Religious (completed years) | | | |  | |  |
| **13** | Occupation of HH head: | | Menial labor (farmer, rickshaw puller, beggar etc ) | | | | 1 | |  |
|  |  |  | Skill (agriculture, job, business etc) | | | | 2 | |  |
| **14** | Amount of land owned by household (decimal): | | Housing and pond | | 0 | 1-9 | | 10+ | |
|  |  |  | Agriculture | | 0 | 1-39 | | 40+ | |

| Interviewer’s name: ---------------------------------------------- | Code -------- | Date of interview -------------- |
| --- | --- | --- |
