# Supplementary material for: Gender differentials in readiness and use of mHealth services in a rural area of Bangladesh
Source: BMC Health Serv Res. 2017 Aug 18;17:573. doi: 10.1186/s12913-017-2523-6 (PMC5563057; doi:10.1186/s12913-017-2523-6)
Supplement: Supplementary file 2 — describes awareness about use of mobile phone for healthcare among males compared to females adjusted for other covariates for age, education and socioeconomic status. Appendix Table S2. describes knowledge of existing mHealth services among males compared to females, adjusted for other covariates for age, education and SES; and Appendix Table S3. describes intention to use mHealth services in future among males compared to females, adjusted for other covariates for age, education and socioeconomic status (DOCX 25 kb) [file 12913_2017_2523_MOESM2_ESM.docx]

Appendix Table 1: Awareness about use of mobile phone for healthcare among males compared to females adjusted for other covariates for age, education and SES in a household survey in rural Bangladesh (N=4,909).

| Variables | Gender | Number of respondents (N) | % of respondents who are aware about mHealth services | OR (95% CI) | p-value |
| --- | --- | --- | --- | --- | --- |
| Gender | Male  Female | 1961  2948 | 38.5  26.5 | 1.7 (1.5–2.0)  1.0 | p<0.001 |
| Age (years) | | | | | |
| 18–29 | Male  Female | 633  1155 | 42.6  35.1 | 1.4 (1.1–1.7)  1.0 | p=0.002 |
| 30–39 | Male  Female | 518  825 | 40.9  27.4 | 1.8 (1.4–2.3)  1.0 | p<0.001 |
| 40–49 | Male  Female | 380  414 | 41.6  21.5 | 2.6 (1.9–3.5)  1.0 | p<0.001 |
| 50+ | Male  Female | 430  554 | 27.0  10.3 | 3.2 (2.3–4.6)  1.0 | p<0.001 |
| Education (years of schooling) | | | | | |
| None | Male  Female | 883  1452 | 28.5  15.1 | 2.2 (1.8–2.8)  1.0 | p<0.001 |
| 1–5 years | Male  Female | 658  804 | 35.9  26.5 | 1.5 (1.2–1.9)  1.0 | p<0.001 |
| 6–10 years | Male  Female | 332  636 | 58.7  46.9 | 1.6 (1.2–2.1)  1.0 | p<0.001 |
| 11+years | Male  Female | 88  56 | 82.9  85.7 | 0.8 (0.3–2.1)  1.0 | p=0.657 |
| Socioeconomic status (asset index) | | | | | |
| Poorest | Male  Female | 330  675 | 24.5  11.8 | 2.4 (1.7–3.4)  1.0 | p<0.001 |
| 2nd | Male  Female | 384  588 | 28.6  19.2 | 1.7 (1.2–2.3)  1.0 | p<0.001 |
| 3rd | Male  Female | 434  566 | 33.6  24.2 | 1.6 (1.2–2.1)  1.0 | p<0.001 |
| 4th | Male  Female | 409  542 | 38.6  30.1 | 1.5 (1.1–1.9)  1.0 | p<0.001 |
| Richest | Male  Female | 404  577 | 64.6  49.4 | 1.9 (1.4–2.4)  1.0 | p<0.001 |

Appendix Table 2: Knowledge of existing mHealth services among males compared to females, adjusted for other covariates for age, education and socioeconomic status in a household survey in rural Bangladesh, N=4,909

| Variables | Gender | Number of respondents (N) | Knowledge of HealthLine 789 | | | Knowledge of government mHealth services at UHC | | |
| --- | --- | --- | --- | --- | --- | --- | --- | --- |
|  |  |  | % of respondents | OR (95% CI) | p-value | % of respondents | OR (95% CI) | p-value |
| Gender | Male  Female | 1961  2948 | 7.04  1.73 | 4.3 (3.1–6.0)  1.0 |  | 7.6  3.1 | 2.5 (1.9–3.2)  1.0 | p<0.001 |
| Age (years) | | | | | | | | |
| 18–29 | Male  Female | 633  1155 | 12.9  3.3 | 4.4 (2.9–6.5)  1.0 | p<0.001 | 9.2  4.8 | 2.0 (1.4–3.0)  1.0 | p<0.001 |
| 30–39 | Male  Female | 518  825 | 6.4  1.5 | 4.6 (2.4–9.0)  1.0 | p<0.001 | 6.4  3.0 | 2.2 (1.3–3.7)  1.0 | p=0.004 |
| 40–49 | Male  Female | 380  414 | 4.2  0.2 | 18.1 (2.4–137.6)  1.0 | p<0.001 | 10.3  2.7 | 4.2 (2.1–8.3)  1.0 | p<0.001 |
| 50+ | Male  Female | 430  554 | 1.6  0.0 | – | – | 4.6  0.5 | 6.7 (2.3–19.8)  1.0 | p<0.001 |
| Education (years of schooling) | | | | | | | | |
| None | Male  Female | 883  1452 | 3.17  0.34 | 9.5 (3.6–24.6)  1.0 | p<0.001 | 5.4  1.1 | 5.2 (2.9–9.1)  1.0 | p<0.001 |
| 1–5 years | Male  Female | 658  804 | 3.9  0.6 | 6.6 (2.5–17.2)  1.0 | p<0.001 | 6.1  2.5 | 2.4 (1.4–4.1)  1.0 | p<0.001 |
| 6–10 years | Male  Female | 332  636 | 14.8  4.1 | 4.1 (2.5–6.7)  1.0 | p<0.001 | 12.9  7.2 | 1.9 (1.2–3.0)  1.0 | p=0.004 |
| 11+ years | Male  Female | 88  56 | 39.8  26.8 | 1.8 (0.9–3.7)  1.0 | p=0.107 | 21.6  21.4 | 1.0 (0.4–2.3)  1.0 | p=0.981 |
| Socioeconomic status (asset index) | | | | | | | | |
| Poorest | Male  Female | 330  675 | 0.0  0.3 | – | – | 4.5  1.4 | 2.9 (1.3–6.3)  1.0 | 0.008 |
| 2nd | Male  Female | 384  588 | 3.85  0.0 | – | – | 4.2  0.8 | 5.1 (1.8–13.9)  1.0 | p<0.001 |
| 3rd | Male  Female | 434  566 | 3.7  0.3 | 10.8 (2.5–47.2)  1.0 | p<0.001 | 5.9  1.4 | 4.4 (2.0–9.9)  1.0 | p<0.001 |
| 4th | Male  Female | 409  542 | 5.6  1.1 | 5.3 (2.1–13.2)  1.0 | p<0.001 | 5.6  3.3 | 1.7 (0.9–3.3)  1.0 | p=0.085 |
| Richest | Male  Female | 404  577 | 21.1  7.3 | 3.4 (2.3–5.0)  1.0 | p<0.001 | 17.3  9.1 | 2.1 (1.4–3.0)  1.0 | p<0.001 |

Appendix Table 3: Intention to use mHealth services in future among males compared to females, adjusted for other covariates for age, education and socioeconomic status in a household survey in rural Bangladesh

| Variables | Gender | Number of respondents (N) | % of respondents | OR (95% CI) | p-value |
| --- | --- | --- | --- | --- | --- |
| Gender | Male  Female | 1961  2944 | 81.4  67.5 | 2.1 (1.8–2.4)  1.0. | <0.001 |
| Age (years) | | | | | |
| 18–29 | Male  Female | 633  1155 | 87.1  69.8 | 2.9 (2.2–3.8)  1.0 | <0.001 |
| 30–39 | Male  Female | 518  825 | 83.4  69.8 | 2.2 (1.6–2.9)  1.0 | <0.001 |
| 40–49 | Male  Female | 380  414 | 79.5  71.3 | 1.6 (1.1–2.2)  1.0 | 0.007 |
| 50+ | Male  Female | 430  554 | 72.3  56.6 | 2.0 (1.5–2.6)  1.0 | <0.001 |
| Education (years of schooling) | | | | | |
| None | Male  Female | 883  1448 | 77.2  62.4 | 2.0 (1.7–2.5) | <0.001 |
| 1–5 years | Male  Female | 658  804 | 82.5  68.9 | 2.1 (1.7–2.7)  1.0 | <0.001 |
| 6–10 years | Male  Female | 332  636 | 87.3  75.6 | 2.2 (1.5–3.2) | <0.001 |
| 11+ years | Male  Female | 88  56 | 92.1  89.3 | 1.4 (0.4–4.4)  1.0 | 0.576 |
| Socioeconomic status (asset index) | | | | | |
| Poorest | Male  Female | 330  673 | 75.1  62.0 | 1.9 (1.4–2.5)  1.0 | <0.001 |
| 2nd | Male  Female | 384  586 | 79.9  65.4 | 2.1 (1.6–2.9)  1.0 | <0.001 |
| 3rd | Male  Female | 434  566 | 80.4  70.3 | 1.7 (1.3–2.3)  1.0 | <0.001 |
| 4th | Male  Female | 409  542 | 81.9  68.8 | 2.0 (1.5–2.8)  1.0 | <0.001 |
| Richest | Male  Female | 404  577 | 88.4  72.3 | 2.9 (2.0–4.1)  1.0 | <0.001 |
